# An Improved Duplex Real-Time Quantitative RT-PCR Assay with a Canine Endogenous Internal Positive Control for More Sensitive and Reliable Detection of Canine Parainfluenza Virus 5

**DOI:** 10.3390/vetsci10020142

**Published:** 2023-02-10

**Authors:** Gyu-Tae Jeon, Hye-Ryung Kim, Yeun-Kyung Shin, Oh-Kyu Kwon, Hae-Eun Kang, Oh-Deog Kwon, Choi-Kyu Park

**Affiliations:** 1 Animal Disease Intervention Center, College of Veterinary Medicine, Kyungpook National University, Daegu 41566, Republic of Korea; 2 Foreign Animal Disease Division, Animal and Plant Quarantine Agency, Gimcheon 39660, Republic of Korea

**Keywords:** canine parainfluenza virus 5, duplex real-time quantitative RT-PCR, L gene, endogenous internal positive control, CIRD

## Abstract

**Simple Summary:**

For reliable detection of canine parainfluenza virus 5, a duplex real-time quantitative RT-PCR assay using the viral L gene and canine *16S rRNA* primers and probe sets was developed in this study. The assay has high analytical sensitivity, specificity, and accuracy. Clinical evaluation results showed that diagnostic sensitivity of the assay was higher than that of the previous HN gene-specific assay and comparable to that of the previous N gene-specific assay. Furthermore, canine *16S rRNA* was stably amplified by the assay in clinical samples, allowing for the avoidance of false negative results. These results suggested that the L gene-specific assay will be a promising tool for the rapid diagnosis and control of canine parainfluenza virus 5 in dogs.

**Abstract:**

A duplex real-time quantitative reverse transcription-polymerase chain reaction (dqRT-PCR) assay was successfully developed to simultaneously detect canine parainfluenza virus 5 (CPIV5) and a canine endogenous internal positive control (EIPC) in canine clinical samples. Two sets of primers and probes for the CPIV5 L and canine 16S rRNA genes were included in the dqRT-PCR assay to detect CPIV and monitor invalid results throughout the qRT-PCR process. The developed dqRT-PCR assay specifically detected CPIV5 but no other canine pathogens. Furthermore, *16S rRNA* was stably amplified by dqRT-PCR assay in all samples containing canine cellular materials. The assay’s sensitivity was determined as below ten RNA copies per reaction, with CPIV5 L gene standard RNA and 1 TCID_50_/mL with the CPIV5 D008 vaccine strain, which was 10-fold higher than that of the previous HN gene-specific qRT-PCR (*HN*-qRT-PCR) assays and was equivalent to that of the previous N gene-specific qRT-PCR (*N*-qRT-PCR) assays, respectively. Moreover, the Ct values of the CPIV5-positive samples obtained using the dqRT-PCR assay were lower than those obtained using the previous *HN*- and *N*-qRT-PCR assays, indicating that the diagnostic performance of the dqRT-PCR assay was superior to those of previous *HN*- and *N*-qRT-PCR assays. The calculated Cohen’s kappa coefficient values (95% confidence interval) between dqRT-PCR and the *HN-* or *N*-specific qRT-PCR assays were 0.97 (0.90–1.03) or 1.00 (1.00–1.00), respectively. In conclusion, the newly developed dqRT-PCR assay with high sensitivity, specificity, and reliability will be a promising diagnostic tool for the detection of CPIV5 in clinical samples and useful for etiological and epidemiological studies of CPIV5 infection in dogs.

## 1. Introduction

Parainfluenza virus 5 (PIV5), taxonomically named the Mammalian *orthorubulavirus*, is a negative-sense nonsegmented single-stranded RNA virus belonging to the genus *Orthorubulavirus* of the subfamily *Rubulavirinae* in the family *Paramyxoviridae*. The PIV5 genome is composed of a 3′ leader region, a 5′ trailer region, and seven nonoverlapping genes: nucleocapsid protein (N), V protein (V), phosphoprotein (P), matrix protein (M), fusion protein (F), small hydrophobic protein (SH), hemagglutinin–neuraminidase protein (HN), and large protein (L) or RNA polymerase genes [1]. PIV5 was first reported in primary monkey kidney cells in 1954 [2], and it is also known as simian virus 5. Since then, PIV5 has frequently been discovered in various hosts, including humans, pigs, dogs, cats, rodents, calves, horses, and lesser pandas [3,4,5,6,7,8], suggesting that the virus is a potential zoonotic pathogen capable of cross-species transmission. In dogs, the canine parainfluenza virus (CPIV) was first identified in laboratory dogs with respiratory disease in 1967. This virus was initially named CPIV type 2 (CPIV2), as it caused respiratory symptoms similar to those caused by human parainfluenza virus type 2 (HPIV2) [9]. However, as subsequent studies revealed that CPIV2 and HPIV2 are antigenically and genetically different, the virus is currently referred to as CPIV5 or CPIV in veterinary medicine [10,11]. The CPIV5 mainly affects the surface epithelium of the respiratory tract, causing mild-to-moderate respiratory illness in dogs. However, severe clinical signs can develop when coinfected with other respiratory viruses or bacteria. Therefore, the virus has been recognized as one of the most important pathogens causing canine infectious respiratory disease (CIRD) worldwide, including in Korea [6,10,11,12,13,14].

Given the clinical impact and worldwide distribution of CPIV5, many studies have been conducted to develop reverse transcription-polymerase chain reaction (RT-PCR)-based assays as rapid and sensitive diagnostic tools for CPIV5 in CIRD-affected clinical samples. Previously, two RT-nested PCR (RT-nPCR) assays operating with three steps (RT, first PCR, and nested PCR) were used to detect N gene CPIV5 by Erles et al. (2004) [15] and Posuwan et al. (2010) [16], respectively. These RT-nPCR assays have been widely used for the surveillance of CPIV5 in CIRD-affected or clinically healthy dogs, either in the original RT-nPCR format or as a modified RT-PCR assay [14,17,18,19]. Moreover, two other two-step RT-PCR assays were developed to detect CPIV5 and used occasionally in China and Japan [20,21]. Although the previously developed classical RT-PCR (cRT-PCR) assays, such as RT-PCR and RT-nPCR, have the advantage of being relatively inexpensive, they have lower sensitivity and specificity, are more time consuming, and are associated with a higher risk of cross-contamination with PCR products than the real-time quantitative RT-PCR (qRT-PCR) assay. In contrast, qRT-PCR assays are relatively expensive, but they have higher sensitivity and specificity, are timesaving, prevent cross-contamination, and have a higher quantification capability of target DNA sequences than the cRT-PCR assays [22,23,24]. To date, the following two types of qRT-PCR assays have been reported for CPIV5: SYBR green-based assay [25,26] and the TaqMan probe-based qRT-PCR assay [27,28]. The SYBR green-based qRT-PCR assay is relatively cost effective and easy to use. However, its specificity is of great concern because a nonspecific dsDNA-binding dye is used to monitor the results of the reaction. The TaqMan probe-based qRT-PCR assay is advisable for a more specific diagnosis of canine pathogens owing to its high specificity mediated by the additional target-specific probe and low susceptibility for visualizing nonspecific PCR products [22,23,24].

The selection of target genes for designing specific primers and probe sets is critical for improving the diagnostic performance of probe-based qRT-PCR assays [24,29]. Two previous studies that described qRT-PCR assays for CPIV5 used HN or N gene-specific primers and probe sets [27,28]. However, it is noteworthy that the previously described qRT-PCR assay for HPIV detection used the L gene-specific primer/probe set and reported that the sensitivity of the assay was higher than that of the HN gene-specific qRT-PCR assay [30]. Moreover, as the L gene is the most conserved viral gene in the family *Paramyxoviridae*, it may be an ideal target for developing a molecular diagnostic assay for paramyxoviruses [31]. However, a qRT-PCR assay targeting the CPIV5 L gene is yet to be reported. Therefore, it is worth trying to design primers and probe sets that target the L gene to secure better diagnostic performance of a new qRT-PCR assay for CPIV5.

Endogenous internal positive controls (EIPC) are commonly used to monitor potential problems that may occur throughout RT-PCR, such as sample collection, transport and storage conditions, nucleic acid extraction, PCR, detection instruments, and potential PCR inhibitors in clinical samples [32]. An appropriate housekeeping gene suitable for the assay should be included as an EIPC to ensure the accuracy and reliability of the assay and minimize false negative test results. For the qRT-PCR assay that screens canine samples for CPIV5, an endogenous internal housekeeping gene stably expressed in canine respiratory clinical specimens should be selected as an EIPC [33,34]. However, to the best of our knowledge, a duplex qRT-PCR assay that can simultaneously amplify both the CPIV5 L gene and EIPC has not been developed for CPIV5 detection in canine clinical samples. In this study, we developed a duplex real-time quantitative RT-PCR (dqRT-PCR) assay, including two sets of primers and probes designed based on the conserved CPIV5 L gene and the canine 16S rRNA gene as an EIPC. The diagnostic performance of the assay was comparatively evaluated with the previously described HN gene-specific qRT-PCR (*HN*-qRT-PCR) and N gene-specific qRT-PCR (*N*-qRT-PCR) assays [27,28] using reference virus strains and clinical samples.

## 2. Materials and Methods

### 2.1. Samples and Nucleic Acid Extraction

A CPIV5 vaccine strain (D008) with a viral titer of ≥10^4.0^ TCID_50_/mL was used to develop and optimize the dqRT-PCR conditions. Two other CPIV5 vaccine strains (Cornell and NL-CPI-5 strains), a Korean field strain detected by the previously reported qRT-PCR assay [27], and seven other canine pathogens were used to evaluate the specificity of the assay. The canine pathogens were as follows: canine respiratory coronavirus (CRCoV, K37 strain), canine coronavirus (CCoV, K378 strain), canine distemper virus (CDV, Onderstepoort strain), canine influenza virus [CIV, A/Canine/Korea/01/07(H3N2)], canine adenovirus 2 (CADV-2, Ditchfield strain), canine parvovirus (CPV, 7809 16-LP strain), and *Bordetella bronchiseptica* (*B.bronchiseptica*, S-55 strain) obtained from commercially available vaccines. All pathogen samples were stored at −80 °C until use. Overall, 266 nasopharyngeal and nasal swab samples and three lung samples were collected from dogs with respiratory clinical signs through the collaboration with a companion animal healthcare company for the clinical evaluation of the dqRT-PCR assay (Postbio Co., Ltd., Guri, Gyeonggi-do, Republic of Korea) and a regional veterinary service laboratory (Daegu, Republic of Korea) from Gyeonggi-do, Gangwon-do, Gyeongsangbuk-do, Gyeongsangnam-do, Chungcheongbuk-do, Chungcheongnam-do, Jeollabuk-do, Jeollanam-do, and Seoul between 2021 and 2022. Before extracting a total nucleic acid from the clinical samples, all samples were immersed into a transport medium (Hank’s Balanced Salt Solution, Sigma-Aldrich, St. Louis, MO, USA) and stored at −80 °C. Further, total nucleic acids were immediately extracted from the collected swab samples. Viral RNA was extracted from each 200 μL sample using a TANBead nucleic acid extraction kit equipped with a fully automated magnetic bead operating platform (Taiwan Advanced Nanotech Inc., Taoyuan, Taiwan). Subsequently, the extract was eluted with 100 μL elution buffer according to the manufacturer’s instructions.

### 2.2. Reference qRT-PCR Assays

The previously reported two qRT-PCR assays were performed using CPIV5 HN or N gene-specific primer and probe sets for a comparative evaluation of developed dqRT-PCR assays as once described with some modifications [27,28]. The *HN*-qRT-PCR assay [28] was performed, including CPIV5 *HN*-qRT-PCR primers (cPIV-1278f and cPIV-1378r) and the probe (cPIV-1320p), using a commercial one-step real-time RT-PCR kit (THUNDERBIRD™ Probe One-step qRT-PCR kit, TOYOBO, Osaka, Japan). Briefly, the qRT-PCR assay was performed in a reaction solution containing 0.5 μL DNA polymerase, 0.5 μL RT Enzyme Mix, 10 μL 2× reaction buffer, 0.4 μM each CPIV5 primer and 0.2 μM probe, and 5 μL RNA. Then, nuclease-free water was added to reach a final volume of 20 μL. The N gene-specific qRT-PCR assay [27] was performed, including CPIV5 N gene-specific primers (NF and NR) and the probe (NP), using a commercial one-step real-time RT-PCR kit (THUNDERBIRD™ Probe One-step qRT-PCR kit, TOYOBO, Osaka, Japan). Briefly, the qRT-PCR assay was performed in a reaction solution containing 0.5 μL DNA polymerase, 0.5 μL RT Enzyme Mix, 10 μL 2× reaction buffer, 0.5 μM each primer and 0.15 μM probe, and 5 μL RNA. Nuclease-free water was added to reach a final volume of 20 μL. The reaction was performed using a CFX96 Touch™ Real-Time PCR Detection System (Bio-Rad, Hercules, CA, USA) according to the manufacturer’s instructions under the following conditions: initial reverse transcription at 50 °C for 10 min, initial denaturation at 95 °C for 1 min, and 40 cycles of thermocycling, including denaturation at 95 °C for 15 s and annealing and extension at 60 °C for 45 s each. The real-time fluorescence values of the FAM-labeled probes were measured in the ongoing reactions at the end of each annealing step. Samples producing a cycle threshold (Ct) of <40 were considered positive to interpret the qRT-PCR results. In contrast, the sample was considered negative when no Ct values were observed during the 40 amplification cycles. The details about the primers and probes used for both qRT-PCR assays are presented in Table 1.

### 2.3. Reference Gene Construction for dqRT-PCR Assay

The partial L gene of CPIV5 spanning the amplified region of each qRT-PCR assay studied was amplified by RT-PCR assay from the CPIV5 D008 vaccine strain using a pair of primers (forward, 5′-GGCAGCATCTTTTTTAACTACT-3′ and reverse, 5′-GTCTCTTGTTCTTTCAAATGGT-3′). These primers were designed based on the sequence of the Korean CPIV5 D277 strain (GenBank accession number KC237065). Moreover, the RT-PCR assay was performed using a commercial RT-PCR kit (Inclone™ one-step RT-PCR kit; Inclone Biotech, Seongnam, Republic of Korea). The reaction mixture (25 μL) comprising 12.5 μL 2× reaction buffer, 0.4 μM each primer, and 5 μL CPIV5 RNA as a template was prepared according to the manufacturer’s instructions. The amplification was conducted in a thermal cycler (Applied Biosystems, Foster City, CA, USA) under the following conditions: reverse transcription at 45 °C for 50 min, initial denaturation at 95 °C for 15 min, 35 cycles of thermocycling, including denaturation at 95 °C for 20 s, annealing at 55 °C for 40 s, extension at 72 °C for 3 min, and a final extension at 72 °C for 5 min. The amplified 460 bp L gene was purified and cloned into the pTOP TA V2 vector using a TOPcloner TA Core Kit (Enzynomics, Daejeon, Republic of Korea). The recombinant plasmid DNA samples were linearized using digestion with EcoRI (TaKaRa Bio, Kusatsu, Japan) and purified using Expin CleanUP SV kit (GeneAll Biotechnology, Seoul, Republic of Korea). Subsequently, in vitro RNA transcription was performed using RiboMAX Large Scale RNA Production System-T7 (Promega Corporation, Fitchburg, WI, USA) according to the manufacturer’s instructions. Further, RNA concentrations were determined by measuring the absorbance at 260 nm using a NanoDrop Lite spectrophotometer (Thermo Fisher Scientific, Waltham, MA, USA). RNA transcript copy numbers were quantified as follows: [copies/μL = plasmid concentration (g/μL)]/[(plasmid length × 340) × (6.022 × 10^23^)]. The RNA transcripts were serially diluted 10-fold (10^7^ to 1 copies/μL) and stored at −80 °C for further use as RNA standards.

### 2.4. Primers and Probe for dqRT-PCR Assay

Two sets of primers and a probe were used for dqRT-PCR to amplify the CPIV5 L gene and canine 16S rRNA housekeeping gene simultaneously. Considering the broad host range of PIV5 and the high genetic homology between strains isolated from different hosts [3,35], L gene sequences of PIV5 strains from different hosts were retrieved from the GenBank database of the National Center for Biotechnology Information (NCBI). The sequences included 12 canine, 7 human, 24 swine, 2 bovine, 1 simian, and 8 other strains (Table S1). Conserved nucleotide sequences within the L genes were identified by multiple alignments using the BioEdit Sequence Alignment Editor program (http://www.mbio.ncsu.edu/BioEdit/ bioedit.html accessed on 11 November 2022). A pair of primers and probe was designed to specifically detect CPIV5 based on these conserved sequences and aided by Geneious Prime (Biomatters Ltd., Auckland, New Zealand). The targeted sequences of the primers (CPIV5-LF and CPIV5-LR) and probe (CPIV5-LP) were aligned with the L gene sequences of PIV5 isolated from other animal hosts (Figure S1A) to determine the specificity of the designed primers and probe.

Furthermore, the potential cross-reactivity of the primers and probe for the CPIV5 L gene was confirmed against random nucleotide sequences using a BLAST search of the NCBI GenBank database (http://www.ncbi.nlm.nih.gov/BLAST/ accessed on 13 November 2022). The canine housekeeping gene 16S rRNA was used as an EIPC marker for the presence of canine cellular materials to avoid false negative results. Eight canine 16S rRNA gene sequences from different breeds (GenBank accession numbers NC002008, MW549038, MW051511, JX088689, CM025140, EU408275, MZ042325, and CM023446) were retrieved from the NCBI GenBank database to design canine 16S rRNA gene-specific primers and probes. Conserved nucleotide sequences within 16S rRNA genes were identified by multiple alignments using the BioEdit Sequence Alignment Editor program. A pair of primers and probes was designed to specifically detect the canine 16S rRNA gene based on these conserved sequences and aided by Geneious Prime software. The specificity of the primers and probe for the canine 16S rRNA gene was confirmed via random nucleotide sequences using a BLAST search on the NCBI GenBank database. For the accurate differential detection of CPIV5 and EIPC using dqRT-PCR, the sequence-specific probes must be labeled with reporter dyes whose fluorescence spectra are distinct or show only minimal overlap [36]. In this study, for the simultaneous and differential detection of the L gene of CPIV5 and EIPC in a single reaction, CPIV5- and EIPC-specific probes were differently labeled at the 5′ and 3′ ends with 6-carboxyfluorescein (FAM) and Black Hole Quencher 1 (BHQ1) for CPIV5, and 6-carboxy-2′,4,4′,5′,7,7′-hexachlorofluorescein (HEX) and BHQ1 for EIPC, according to the manufacturer’s guidelines (BIONICS, Daejeon, Republic of Korea) (Table 1).

### 2.5. Optimization of dqRT-PCR Conditions

Before optimizing the dqRT-PCR assay, a monoplex qRT-PCR assay was performed with each CPIV5 or EIPC primers and probe combination using a commercial qRT-PCR kit (THUNDERBIRD™ Probe One-step qRT-PCR kit, TOYOBO, Osaka, Japan) and a CFX96 Touch™ Real-Time PCR Detection System (Bio-Rad, Hercules, CA, USA). The 20 μL of reaction mixture contained 0.5 μL of DNA Polymerase, 0.5 μL of RT Enzyme Mix, 10 μL of 2× reaction buffer, 0.4 μM of each primer and 0.2 μM probe, and 5 μL CPIV5 standard RNA for CPIV5 or CPIV5-negative canine RNA for an EIPC, prepared according to the manufacturer’s instructions. To set optimal dqRT-PCR conditions, the concentrations of the two sets of primers and probe were optimized, whereas the other reaction components were kept the same as those used for monoplex qRT-PCR. Moreover, the monoplex and duplex qRT-PCR programs were used under the following conditions: the reaction was conducted in a CFX96 Touch™ Real-Time PCR Detection System (Bio-Rad, Hercules, CA, USA) with initial reverse transcription at 50 °C for 10 min, initial denaturation at 95 °C for 1 min, 40 cycles of denaturation at 95 °C for 15 s, and annealing and extension at 60 °C for 45 s, as previously described and according to the manufacturer’s instructions. Notably, the FAM (CPIV5) and HEX (EIPC) fluorescence signals for the tested samples were measured at the end of each annealing step in each cycle. The Ct values for each sample were calculated by determining the point at which the fluorescence exceeded the threshold limit. To interpret the dqRT-PCR results, samples with Ct values of <40 were considered positive. In contrast, the sample was considered negative when no Ct values were observed during the 40 amplification cycles.

### 2.6. Sensitivity of dqRT-PCR Assays

The analytical sensitivity of the dqRT-PCR assay for CPIV5 RNA was determined in triplicate using serial dilutions (10^6^–10^0^ copies/reaction) of the standard CPIV5 L gene RNA. The dqRT-PCR assay was performed with mixed RNA templates extracted from CPIV5-negative canine oropharyngeal swabs or lung samples spiked with serial dilutions of the CPIV5 standard RNAs described above to confirm the interference in amplification and detection between the target CPIV5 RNA and EIPC. CFX96 Touch™ Real-Time PCR Detection software (Bio-Rad, Hercules, CA, USA) was used to create a standard curve with Ct values of 10-fold dilutions of CPIV5 RNA for data analysis. The correlation coefficient (*R*^2^) of the standard curve, standard deviations of the results, and CPIV5 copy numbers in the samples were calculated using detection software based on the standard curves. In order to improve the accuracy of limits of detection (LOD), the LOD95% of the dqRT-PCR, 20-iterations of dqRT-PCR were conducted using 5 concentrations of standard RNAs (8, 6, 4, 2, and 0.5 copies/reaction). The data were analyzed by Analyse-it program (Microsoft, Redmond, WA, USA) according to the manufacturer’s instructions. Additionally, the sensitivity of the dqRT-PCR assay was determined in triplicate using 10-fold serial dilutions of RNAs extracted from the CPIV5 D008 vaccine strain showing a viral titer of ≥10^4.0^ TCID_50_/mL and the results were compared with those of the previously described *HN*- and *N*-qRT-PCR assays [27,28].

### 2.7. Specificity of dqRT-PCR Assays

Total nucleic acids were extracted from three CPIV5 vaccine strains (D008, Cornell, and NL-CPI-5), a CPIV5 Korean isolate strain, seven other canine pathogens (CRCoV, CCoV, CDV, CIV, CADV-2, CPV, and *B.bronchiseptica*), and two CPIV5-negative canine clinical samples, which were confirmed by the previously described qRT-PCR assays [27,28], to test the specificity of the dqRT-PCR assay. Moreover, one canine-origin cell culture (MDCK cells) and two non-canine-origin cell cultures (Vero and ST cells) were used as negative controls (Table 2).

### 2.8. Precision of dqRT-PCR Assays

The repeatability (intra-assay precision) and reproducibility (inter-assay precision) of the dqRT-PCR assay for CPIV5 were determined using three different concentrations (high, medium, and low) of each tested viral standard gene. The concentrations of the L genes in CPIV5 were 10^6^, 10^4^, and 10^2^ copies/reaction, respectively. Intra-assay variability was determined by analyzing each dilution in triplicate on the same day, and inter-assay variability was determined by analyzing each dilution in six independent experiments performed by two different operators on different days, per the MIQE guidelines [23]. The coefficient of variation (CV) for the Ct values was determined using the result of the intra-assay or inter-assay experiments and expressed as a percentage of the mean value alongside the standard deviation values.

### 2.9. Clinical Evaluation of dqRT-PCR Assay

For clinical evaluation, 269 clinical samples (266 nasopharyngeal swab samples and three lung samples) were collected and tested using the newly developed dqRT-PCR assay. Samples were considered positive if both fluorescence signals of the CPIV5 L gene and EIPC were detected, negative if only EIPC was amplified, and invalid if EIPC was not amplified. The dqRT-PCR results were compared with those of the HN gene- and N gene-specific qRT-PCR assays [27,28]. Inter-assay concordance was analyzed using Cohen’s kappa statistic at a 95% confidence interval (CI). The interpretation of the calculated kappa coefficient value (κ) was as follows: κ < 0.20 = slight agreement, 0.21–0.40 = fair agreement, 0.41–0.60 = moderate agreement, 0.61–0.80 = substantial agreement, and 0.81–1.0 = almost perfect agreement [37]. If there were discrepant results between assays, the target viral gene of the assay was amplified using RNAs obtained from a discordant sample as described above and sequenced using Sanger’s method by a commercial sequencing company (BIONICS, Daejeon, Republic of Korea). This procedure was performed to determine whether the cause of the discrepancy between the assays was due to a mismatch between the primers/probe and the viral target gene sequences. SYBR green-based qRT-PCR assay was conducted to further analyze the discordant samples using the same reaction conditions as the original qRT-PCR assay, except that the probe was removed and SYBR green intercalating dye was added to the reaction mixture.

## 3. Results

### 3.1. Interpretation of the dqRT-PCR Assay

**Figure 1:**
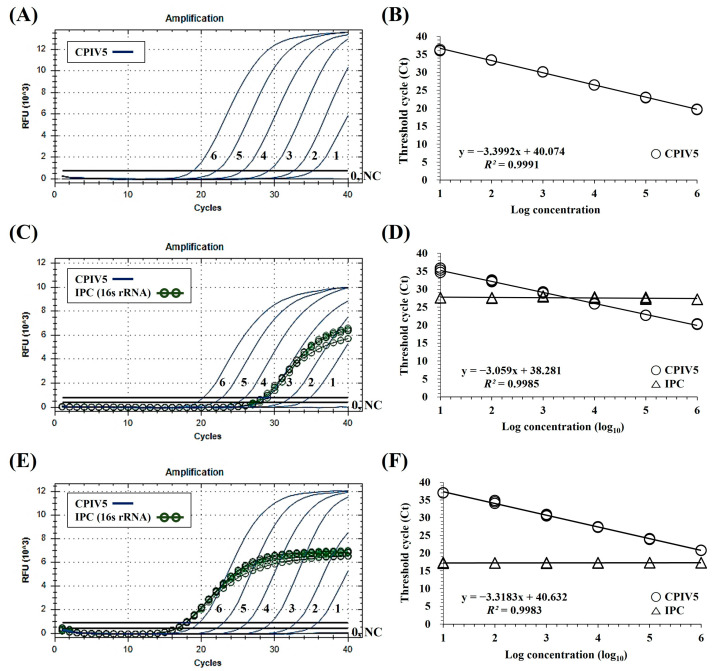
The limit of detection (LOD) and standard curve of duplex real-time quantitative reverse transcription-polymerase chain reaction (dqRT-PCR) assay. LOD and a standard curve of the assay with 10-fold serial dilutions of canine parainfluenza virus 5 (CPIV5) standard RNAs (**A**,**B**), 10-fold serial dilutions of CPIV5 standard RNAs spiked into canine nasopharyngeal swab samples (**C**,**D**), and lung samples (**E**,**F**). Lines 6–0, 10-fold serial dilutions of the CPIV5 standard RNAs (10^6^–10^0^ copies/reaction). The coefficient of determination (*R*^2^) and the equation of the regression curve (y) were calculated using the CFX Manager Software (Bio-Rad).

The fluorescent signals of FAM for CPIV5 and HEX for EIPC were successfully generated by dqRT-PCR assay, with each set of primers and probe corresponding to CPIV5 RNA or canine RNA samples, respectively (Figure 1). The results of the dqRT-PCR assay using the optimized concentration of primers and probes (0.4 μM each primer and 0.2 μM of each probe for CPIV5 and EIPC, respectively) showed that two fluorescent signals of FAM and HEX could be generated simultaneously in a single reaction. Additionally, HEX signals for EIPC were consistently generated in different types of samples, regardless of the concentration of CPIV5 standard RNA (Figure 1C,E). These results demonstrated that the dqRT-PCR assay can successfully amplify the two target genes of CPIV5 and canine *16S rRNA* in clinical samples in a single reaction without spurious amplification or significant cross-reactivity between the two fluorescent dyes.

### 3.2. Specificity and Sensitivity of the dqRT-PCR Assay

The established dqRT-PCR assay, including primers and probe set for CPIV5, detected all four CPIV5 strains, and no positive signals were obtained for seven other canine pathogens and five negative controls (including two CPIV5-negative canine clinical samples and three cell cultures). Positive signals were detected in all canine pathogen samples, two canine clinical samples, and a canine-origin cell culture (MDCK cell), except for CCoV, CRCoV, and two non-canine cell cultures in the dqRT-PCR assay, including primers and probe for EIPC (Table 2). These results demonstrated that the established dqRT-PCR assay was highly specific to CPIV5 and was considered reliable as it could prevent false negative results. The LOD of dqRT-PCR assay was below ten copies/reaction for CPIV5 (Figure 1). Furthermore, standard curves were generated for the targeted genes by plotting their Ct values against their dilution factors to assess the PCR efficiency and linearity of the reaction. The dqRT-PCR assay revealed a high correlation value (*R*^2^ > 0.99) between the Ct values and dilution factors (Figure 1). In addition, the result of LOD95% of dqRT-PCR was 9.517 copies with a 95% CI interval (5.866 to 15.44) and *p* value was <0.0001 (Table S3). Given that the target gene sequences of each assay were different, the sensitivity of the dqRT-PCR assay, including L gene-specific primers and probe, was further evaluated with 10-fold serial dilutions of viral nucleic acids (from 10^0^ to 10^−5^ dilution) extracted from the CPIV5 D008 vaccine strain with a viral titer of ≥10^4.0^ TCID_50_/mL, and the results were compared with those of previous *HN*- and *N*-qRT-PCR assays [27,28] using the same RNA templates. Positive results of the developed dqRT-PCR assays were observed at dilutions ranging from 10^4^ to 10^−1^ TCID_50_/mL. We found that the sensitivity of the dqRT-PCR assay (1 TCID_50_/mL) was 10 times higher than that of the *HN*-qRT-PCR (10 TCID_50_/mL) and was equivalent to that of the *N*-qRT-PCR assay (1 TCID_50_/mL) (Table 3).

### 3.3. Precision of the dqRT-PCR Assay

Three different concentrations of CPIV5 standard RNA samples were tested in triplicate in six different runs performed by two operators on different days to assess intra-assay repeatability and inter-assay reproducibility. The coefficients of variation within (intra-assay variability) and between (inter-assay variability) runs were 0.1–1.2% and 1.4–2.0%, respectively (Table 4). These results indicate that the dqRT-PCR assay developed in this study can be used as an accurate and reliable diagnostic tool for CPIV5.

### 3.4. Comparative Clinical Evaluation of the dqRT-PCR Assay

**Figure 2:**
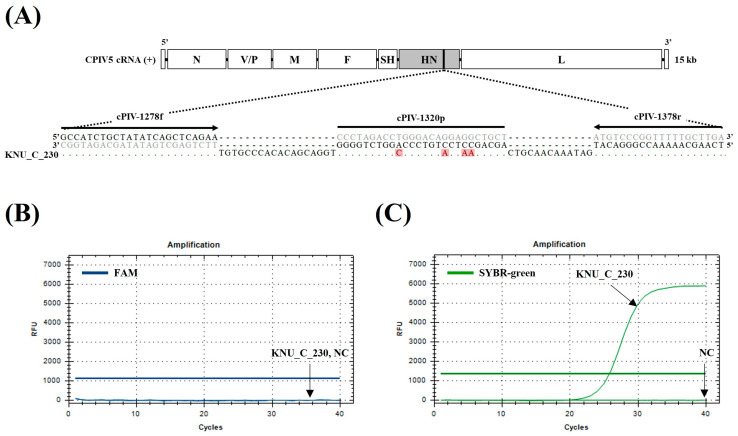
Alignment of canine parainfluenza virus 5 HN gene sequence obtained from a discordant sample with primers and probe-binding sequences of the previously reported HN gene-specific real-time quantitative reverse transcription polymerase chain reaction (*HN*-qRT-PCR) assay, and diagnostic results of the sample by *HN*-qRT-PCR assays, with and without a probe. (**A**) Four mismatches were identified in the primers and probe-binding sites of the *HN*-qRT-PCR assay. The identical bases were denoted by a dot, and the mismatched bases were denoted by a letter with a red background color. The black arrow lines and a blunt line indicate primers and probe sequences, respectively. Reverse primers are reverse complemented. (**B**,**C**) Diagnostic results of the discordant sample by original *HN*-qRT-PCR assay with a target gene-specific probe (FAM) (**B**) and modified SYBR green-based *HN*-qRT-PCR without the probe (**C**). KNU_C_230, sample ID of discordant sample; NC, negative control.

The dqRT-PCR assay detected 16 out of 269 clinical samples as CPIV5-positive and canine *16S rRNA* (EIPC) was successfully amplified in all clinical samples, except one. The detection rate of CPIV5 by the dqRT-PCR assay was 5.9%, which was higher than that of the *HN*-qRT-PCR assay (5.6%) and the same as that of the *N*-qRT-PCR assay (5.9%) (Table 5). The range of Ct values for CPIV5-positive samples was as follows: 16.19–33.63 using the dqRT-PCR assay, 21.50–39.10 using the *HN*-qRT-PCR assay, and 18.89–38.39 using the *N*-qRT-PCR (Table S2). The Ct values produced by the dqRT-PCR assay were lower than those produced by the *HN*- and *N*-qRT-PCR assays, indicating that the dqRT-PCR assay was more sensitive than the previously reported *HN*- and *N*-qRT-PCR assays for the detection of CPIV5 RNAs from clinical samples. The percentages of positive, negative, and overall agreements between the results of the dqRT-PCR and the *HN*- or *N*-qRT-PCR assays were 93.8% (15/16), 100.0% (253/253), and 99.6% (268/269) with the κ values of 0.97 (0.90–1.03), 100.0% (16/16), 100.0% (253/253), and 100.0% (269/269) with the κ value of 1.00, respectively. It was indicated by these results that the diagnostic results of the dqRT-PCR almost perfectly agreed with those of the *HN*-qRT-PCR and perfectly agreed with those of the *N*-qRT-PCR (Table 5). In this study, the *HN*-qRT-PCR assay failed to detect CPIV5 RNA from one nasopharyngeal swab sample that was CPIV5-positive by both dqRT-PCR and *N*-qRT-PCR assays (Table 5 and Table S2). To elucidate the cause of the misdiagnosis for the discordant sample, the target gene fragment was amplified using the sequencing primers (forward primer 5′-TCTCGCTCACCAAGACAC-3′; backward primer 5′-GCTGGGTCAGACCTGGA-3′) containing the binding sites for the *HN*-qRT-PCR primers and probe. The sequence was analyzed using the Sanger method by a commercial company (BIONICS, Daejeon, Republic of Korea). The sequence analysis result revealed that there were four mismatches in the probe binding site (the 6th, 7th, 10th, and 17th bases from the 5′ end of the probe) of the HN gene sequences, whereas there were no mismatches in the primer binding sites (Figure 2A). Furthermore, to determine whether the detection failure of the *HN*-qRT-PCR was due to sequence mismatches of the probe binding site, the discordant sample was retested by SYBR Green-based qRT-PCR under the same reaction conditions as the *HN*-qRT-PCR, except that the mismatched probe was excluded. Surprisingly, CPIV5 RNA was successfully amplified from the discordant sample by SYBR green-based qRT-PCR assay, with a Ct value of 25.81 (Figure 2C). This result indicated that the *HN*-qRT-PCR assay for the discordant sample was a false negative result caused by sequence mismatches in the probe binding site.

## 4. Discussion

Globally, CPIV5 is one of the most frequently identified viral pathogens in dogs suffering from CIRD. Therefore, several RT-nPCR [15,16], cRT-PCR [14,17,18,19,20,21,26], and qRT-PCR [27,28] assays have been developed and used for sensitive and specific detection of the virus from the suspected canine specimens. Among these, a qRT-PCR assay using a TaqMan probe is more desirable for detecting CPIV5 because it is more sensitive and specific than RT-nPCR and cRT-PCR assays. In addition, the cross-contamination of pre-amplified DNA may be prevented by eliminating any post-PCR analysis [22,24]. To date, only two qRT-PCR assays have been reported for detecting the CPIV5 HN [28] and N [27] genes. The *HN*-qRT-PCR assay, including HN gene-specific primers and probe, was reported by Windsor et al. (2006) [28], but the analytical and diagnostic performances were not fully validated. Meanwhile, the *N*-qRT-PCR with N gene-specific primers and probe was recently reported by Dong et al. (2022) [27], and the analytical and diagnostic performance was fully validated using standard RNAs and clinical samples. However, since the two qRT-PCR assays have been only used in the study in which it was reported, and there is no report comparing the diagnostic performance of the two assays, it was impossible to determine which assay is more suitable for diagnosing CPIV5 in clinical samples. Furthermore, a qRT-PCR assay targeting the CPIV5 L gene has not been developed to date despite the fact that the most conserved L gene may be an ideal target for developing a molecular diagnostic assay for paramyxoviruses [31,35]. Therefore, we newly developed a dqRT-PCR assay in which the CPIV5 L gene and canine 16S rRNA gene can be simultaneously detected. The diagnostic performance of the assay was comparatively evaluated with the previously reported *HN*- and *N*-qRT-PCR assays for the accurate and reliable detection of CPIV5 in canine clinical specimens.

Given the broad host range and the cross-species transmission of the PIV5 [3,4,5,6,7,8,38,39], the primers and probe set for the dqRT-PCR assay developed in this study was carefully designed to detect CPIV5 and PIV5 strains from different non-canine hosts based on the RNA-dependent RNA polymerase-encoding region of the PIV5 L gene, which is the most conserved viral gene in PIV5 strains isolated from different hosts [35]. Notably, the sequences of the designed primers and probe of the dqRT-PCR assay had no mismatches on the target gene sequences of all available PIV5 strains from different hosts (Figure S1A). Moreover, we further designed a set of primers and probe that could amplify the canine 16S rRNA gene as an EIPC in order to avoid false negative results by unsuitable sampling, nucleic acid extraction, and the RT-PCR process. The results of the developed dqRT-PCR assay revealed that the newly designed L gene-specific primers and probe successfully amplified four CPIV5 strains, but not the other seven canine pathogens, suggesting that the primers and probe were highly specific to CPIV5 (Table 2). Moreover, the analytical sensitivity of the developed dqRT-PCR assay was 10 times higher than that of the previous *HN*-qRT-PCR assay [28] and comparable to that of the previous *N*-qRT-PCR assay [27] (Table 3). The performance of canine *16S rRNA* (EIPC) in dqRT-PCR assay was evaluated by analytical analysis, indicating that EPIC does not interact with CPIV5 targets. No effect was observed on the amplification efficiency and sensitivity of the assay for CPIV5 detection (Figure 1).

High repeatability and reproducibility were observed in the repeatability and reproducibility tests, with coefficients of intra- and inter-assay variation of <2.0%. These results indicate that the dqRT-PCR assay developed in this study can be an accurate and reliable diagnostic tool for CPIV5 (Table 4).

Subsequently, evaluation of 269 canine clinical samples revealed that the detection rate of CPIV5 by the developed dqRT-PCR assay (5.9%) was higher than that of previously reported HN gene-specific qRT-PCR assay (5.6%) and consistent with that of N gene-specific qRT-PCR assay (5.9%) (Table 5). Furthermore, canine *16S rRNA* (EIPC) was amplified using dqRT-PCR assay in all tested canine clinical samples, except for one. Therefore, it was possible to filter out invalid samples and ensure the high reliability of the developed dqRT-PCR assay. When comparing the diagnostic results of the assays, one nasopharyngeal swab sample (KNU_C_230) that was CPIV5 positive in both dqRT-PCR and *N*-qRT-PCR assays was determined CPIV5 negative by the *HN*-qRT-PCR. Furthermore, analysis using target gene sequencing and SYBR green-based qRT-PCR assay revealed that the discordant sample was confirmed as true positive for CPIV5. In addition, the misdiagnosis of the samples was caused by mismatches of the probe binding site at the target HN gene sequences (Figure 2). According to previous studies, sequence mismatches between the target gene and the primers and probe may decrease the sensitivity of the qPCR assay and lead to false negative results in clinical samples with a low amount of the target pathogen [40].

Additionally, it has been reported that the effect of mismatches in the probe binding site is more severe on the sensitivity of a qPCR assay than on the primer binding site [40,41,42,43]. Although both primer sequences of the *HN*-qRT-PCR assay were concordant with most PIV5 strains, the probe sequence was mismatched with multiple false-proving sites (Figure S1B). Moreover, four mismatched bases were identified in the probe binding site of the HN gene sequences obtained from the discordant clinical sample. This false negative result from the *HN*-qRT-PCR assay may be attributed to the incompatibility of the probe with the sequences of some CPIV5 strains, including the Korean field strain evaluated in this study (Figure 2A and Figure S1B). In contrast, the sequences of the primers and probe of the *N*-qRT-PCR were almost concordant with the target N gene sequences of the PIV5 strains, although one nucleotide mismatch was found in the primer or probe binding sites of some PIV strains (Figure S1C). When comparing the Ct values of CPIV5-positive samples for clinical evaluation, the delayed Ct values of the *HN*- and *N*-qRT-PCR assays compared with those of the dqRT-PCR assay are most likely caused by these mismatches of the probe or primer binding site (Table S2 and Figure S1). Therefore, redesigning the probes and/or primers used in these assays is necessary to ensure maximum diagnostic performance for the detection of genetically diverse PIV strains.

Based on the diagnostic results of dqRT-PCR for the 269 canine clinical samples, the prevalence of CPIV5 was 5.9% (Table 5). The prevalence of CPIV5 in this study (5.9%) was lower than that in a previous study in Korea for stray dogs (13.1%) [44], but it was similar to those of previous studies in Korea for hospitalized and sheltered dogs (2.0–11.1%) [45,46]. Moreover, the prevalence of CPIV5 in this study (5.9%) was higher than that in the USA (1.5%) [27] and Japan (1.8%) [25], but significantly lower than that in Thailand (35.3%) [19]. Various factors, such as the country and region surveyed, health status of the tested dogs, sample collection period, and diagnostic method, could explain the differences in the observed prevalence. Therefore, the data obtained in this study contribute to expanding our knowledge about the current infection status and epidemiology of CPIV5 in the Korean dog population.

In this study, we newly developed a dqRT-PCR assay that simultaneously amplifies the CPIV5 L gene and the canine 16S rRNA gene (EIPC) in a reaction. The diagnostic performance of the dqRT-PCR assay was superior to those of the previously reported *HN*- and *N*-qRT-PCR assays. Moreover, this assay secured high diagnostic reliability by incorporating the canine 16S rRNA gene as an EIPC. The dqRT-PCR assay, with high sensitivity, specificity, and reliability, will be a promising diagnostic tool for CPIV5 in clinical samples and contribute to expanding the knowledge about the etiology and epidemiology of CPIV5 infection in dogs.

## Figures and Tables

**Table 1:** Primers and probes used in this study.

Method	Target Gene	Primer and Probe	Primer/Probe Sequence (5′–3′) ^a^	Genome Position ^b^	Amplicon Size (bp)	Reference
dqRT-PCR	*L*	CPIV-LF	GCTTGGACAATGATATCTATCTC	10649–10671	83	This study
		CPIV-LR	TCTCCCTGCACCATACTC	10714–10731		
		CPIV-LP	FAM–CCACAGAGTCTGGCACACGAGTA–BHQ1	10689–10711		
	Canine *16S rRNA*	EIPC-F	AGACGAGAAGACCCTATG	2142–2159	116	This study
		EIPC-R	GGTCACCCCAACCTAAAT	2240–2257		
		EIPC-P	HEX–ACCTACAAGGCATAACATAACACCA–BHQ1	2199–2223		
qRT-PCR	*HN*	cPIV-1278f	GCCATCTGCTATATCAGCTCAGAA	7861–7884	101	Windsor et al. [28]
		cPIV-1378r	TCAAGCAAAAACCGGGACAT	7942–7961		
		cPIV-1320p	FAM–AGCAGCCTCCTGTCCCAGGTCTAGGG–BHQ1	7903–7928		
	*N*	NF	GATCATTCCGCTTAATCCCC	480–499	77	Dong et al. [27]
		NR	TTCTGCAAGTGCAGCATAGG	537–556		
		NP	FAM–TCGTTCAGGTATGAGCCGTGGA–BHQ1	505–526		

^a^ FAM, 6-carboxyfluorescein; HEX, 6-carboxy-2′,4,4′,5′,7,7′-hexachlorofluorescein; BHQ1, Black Hole Quencher 1. ^b^ Locations of all primer and probe sequences for the monoplex real-time quantitative reverse transcription-polymerase chain reaction (qRT-PCR) and duplex qRT-PCR (dqRT-PCR) assays. The assays were derived from the complete genome sequence of the canine parainfluenza virus 5 (CPIV5) CPI+ strain (GenBank accession number JQ743321.1) and canine 16S rRNA gene (GenBank accession number MZ042325).

**Table 2:** Specificity of duplex real-time quantitative reverse transcription-polymerase chain reaction assay, including canine parainfluenza virus 5 (CPIV5) and canine endogenous internal positive control (EIPC) specific primers and probe sets.

Pathogen	Strain	Source ^a^	Amplification of Target Gene	
			CPIV5 (FAM)	EIPC (HEX)
Canine parainfluenza virus 5	D008	CAVS	+	+
Canine parainfluenza virus 5	Cornell	CAVS	+	+
Canine parainfluenza virus 5	NL-CPI-5	CAVS	+	+
Canine parainfluenza virus 5	Korean isolate	ADIC	+	+
Canine respiratory coronavirus	K37	APQA	−	−
Canine coronavirus	K378	CAVS	−	−
Canine distemper virus	Onderstepoort	CAVS	−	+
Canine influenza virus	A/Canine/Republic of Korea/01/07(H3N2)	CAVS	−	+
Canine adenovirus 2	Ditchfield	CAVS	−	+
Canine parvovirus	7809 16-LP	CAVS	−	+
*Bordetella bronchiseptica*	S-55	CAVS	−	+
Non-infected canine swab sample	-	APQA	−	+
Non-infected canine lung sample	-	APQA	−	+
MDCK cell	-	ADIC	−	+
ST cell	-	ADIC	−	−
Vero cell	-	ADIC	−	−

^a^ CAVS, commercially available vaccine strain; APQA, Animal and Plant Quarantine Agency, Republic of Korea; ADIC, Animal Disease Intervention Center, Kyungpook National University, Republic of Korea. +: positive reaction; −: negative reaction.

**Table 3:** Comparison of sensitivity between the developed duplex real-time quantitative reverse transcription-polymerase chain reaction (dqRT-PCR) and previously reported monoplex qRT-PCR assays.

Dilutions (TCID_50_/mL)	Results of Different Assays ^a^								
	L gene-Specific dqRT-PCR			HN Gene-Specific qRT-PCR			N Gene-Specific qRT-PCR		
	1-Folds	2-Folds	3-Folds	1-Folds	2-Folds	3-Folds	1-Folds	2-Folds	3-Folds
10^4^	21.49	21.53	21.52	22.10	22.74	22.49	21.79	21.87	21.86
10^3^	24.75	24.97	24.94	25.82	26.08	26.19	25.14	25.37	25.29
10^2^	28.03	28.10	28.16	29.59	29.85	30.03	28.50	28.28	28.35
10^1^	31.26	31.46	34.35	32.99	33.53	33.17	31.39	31.33	31.47
10^0^	34.95	33.97	34.11	37.75	NC	36.85	34.94	34.32	34.45
10^−1^	NC	NC	NC	NC	NC	NC	NC	NC	NC

^a^ The sensitivities of the developed duplex real-time quantitative reverse transcription-polymerase chain reaction (dqRT-PCR) and previously reported HN gene- and N gene-specific monoplex qRT-PCR assays were determined in triplicate using 10-fold serial dilutions of RNAs extracted from the CPIV5 D008 vaccine strain showing a viral titer of ≥10^4.0^ TCID_50_/mL and the results were compared. NC: No Ct value.

**Table 4:** Intra- and inter-assay coefficient of variation of the duplex real-time quantitative reverse transcription-polymerase chain reaction (dqRT-PCR).

Concentration of RNA (Copies/Reaction)	Ct Values of the dqRT-PCR for CPIV5 RNAs					
	Intra-Assay			Inter-Assay		
	Mean	SD	CV (%)	Mean	SD	CV (%)
High (10^6^)	20.73	0.03	0.1	20.33	0.29	1.4
Medium (10^4^)	27.34	0.11	0.4	26.60	0.51	1.9
Low (10^2^)	34.45	0.42	1.2	33.40	0.66	2.0

The mean value, standard deviation (SD), and coefficient of variation (CV) were determined based on the Ct values for dqRT-PCR with canine parainfluenza virus 5 (CPIV5) RNA standards.

**Table 5:** Comparative diagnostic results for the detection of canine-parainfluenza virus 5 in clinical samples.

Test Results of Different Assays		New dqRT-PCR			Detection Rate	Overall Agreement
		Positive	Negative	Total		
HN gene-specific qRT-PCR	Positive	15	0	15	5.6%	99.6%
	Negative	1	253	254		
	Total	16	253	269		
N gene-specific qRT-PCR	Positive	16	0	16	5.9%	100.0%
	Negative	0	253	253		
	Total	16	253	269		
Detection rate		5.9%				

The percentages of positive, negative, and general agreement among the results of the newly developed duplex real-time quantitative reverse transcription-polymerase chain reaction (dqRT-PCR) and the previously reported HN gene- or N gene-specific monoplex qRT-PCR assays were 93.8% (15/16), 100.0% (253/253), and 99.6% (268/269) or 100.0% (16/16), 100.0% (253/253), and 100.0% (269/269), respectively. The calculated kappa coefficient values (95% confidence interval) between dqRT-PCR and the HN gene- or N gene-specific qRT-PCR assays were 0.97 (0.90–1.03) or 1.00 (1.00–1.00), respectively.

## Data Availability

Not applicable.

## Supplementary Materials

The following supporting information can be downloaded at: https://www.mdpi.com/article/10.3390/vetsci10020142/s1, Figure S1: Alignment of sequences between primers/probe of the L, HN, and N gene-specific qRT-PCR assays and the corresponding target genes of canine parainfluenza virus 5 (CPIV5) isolated from different hosts; Table S1: Genome sequences of PIV5 strains collected from the GenBank database; Table S2: Comparison of diagnostic results for 16 canine parainfluenza virus 5-positive clinical samples by the newly developed L gene-specific duplex real-time quantitative reverse transcription PCR (dqRT-PCR) assay and previously reported HN gene-specific and N gene-specific qRT-PCR assays; Table S3: The result of LOD95% of dqRT-PCR.
