# Supplementary material for: An Improved Duplex Real-Time Quantitative RT-PCR Assay with a Canine Endogenous Internal Positive Control for More Sensitive and Reliable Detection of Canine Parainfluenza Virus 5
Source: Vet Sci. 2023 Feb 10;10(2):142. doi: 10.3390/vetsci10020142 (PMC9965950; doi:10.3390/vetsci10020142)
Supplement: Supplementary file 1 [file vetsci-10-00142-s001.zip › Supplementary Figure S1.pdf]

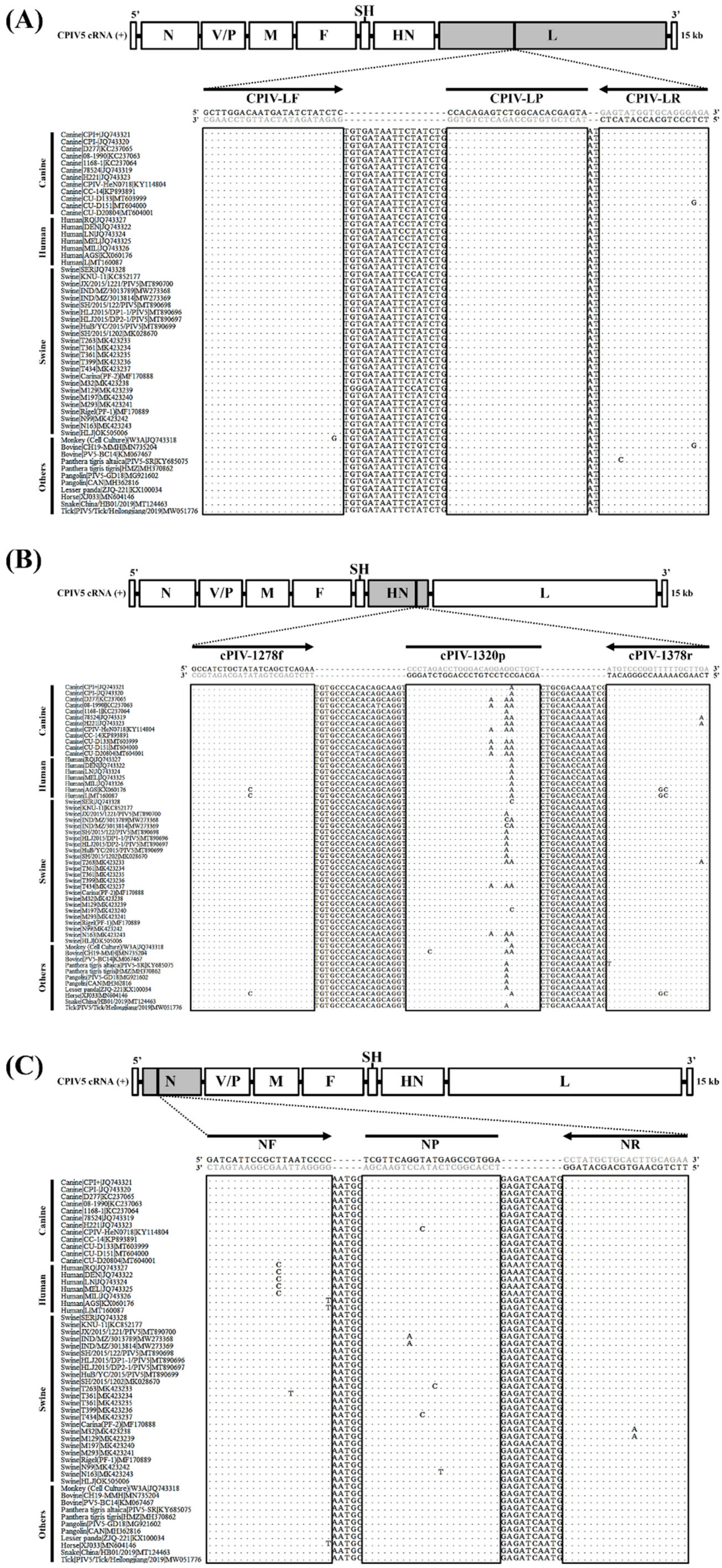

(A)
CPIV5 cRNA (+)
5'
N
V/P
M
F
SH
HN
L
3'
15 kb
CPIV-LF
CPIV-LP
CPIV-LR
Canine
Human
Swine
Others
(B)
CPIV5 cRNA (+)
5'
N
V/P
M
F
SH
HN
L
3'
15 kb
cPIV-1278f
cPIV-1320p
cPIV-1378r
Canine
Human
Swine
Others
(C)
CPIV5 cRNA (+)
5'
N
V/P
M
F
SH
HN
L
3'
15 kb
NF
NP
NR
Canine
Human
Swine
Others

Figure S1. Alignment of sequences between primers/probe of the L, HN, and N gene-specific qRT-PCR assays and the corresponding target genes of canine parainfluenza virus 5 (CPIV5) isolated from different hosts. (A, B, and C) Sequence variations in primers and probe binding regions for developed L gene-specific dqRT-PCR, the previously reported HN gene-specific-, and N gene-specific-qRT-PCR assays, respectively. The black arrow lines and the blunt line indicate primers and probe, respectively. A dot indicates an identical nucleotide, and a letter indicates a different base between primers/probes and the targeted gene sequences of PIV5 strains. Reverse primers are reverse complemented.
