# Supplementary material for: An Improved Duplex Real-Time Quantitative RT-PCR Assay with a Canine Endogenous Internal Positive Control for More Sensitive and Reliable Detection of Canine Parainfluenza Virus 5
Source: Vet Sci. 2023 Feb 10;10(2):142. doi: 10.3390/vetsci10020142 (PMC9965950; doi:10.3390/vetsci10020142)
Supplement: Supplementary file 1 [file vetsci-10-00142-s001.zip › Supplementary Table S1.pdf]

Table S1. Genome sequences of PIV5 strains collected from the GenBank database.

No.	Host	Strain	Collected Country	Collected year	GenBank accession number
1	Canine	CPI+	United States	1980	JQ743321
2	Canine	CPI-	United States	1980	JQ743320
3	Canine	D277	South Korea	2008	KC237065
4	Canine	08-1990	South Korea	2009	KC237063
5	Canine	1168-1	South Korea	2009	KC237064
6	Canine	78524	United Kingdom	2014	JQ743319
7	Canine	H221	United Kingdom	2014	JQ743323
8	Canine	CPIV-HeN0718	China	2015	KY114804
9	Canine	CC-14	China	2015	KP893891
10	Canine	CU-D133	Thailand	2016	MT603999
11	Canine	CU-D151	Thailand	2016	MT604000
12	Canine	CU-D20804	Thailand	2018	MT604001
13	Human	RQ	United Kingdom	1976	JQ743327
14	Human	DEN	United Kingdom	1980	JQ743322
15	Human	LN	United Kingdom	1980	JQ743324
16	Human	MEL	United Kingdom	1980	JQ743325
17	Human	MIL	United Kingdom	1980	JQ743326
18	Human	AGS	United States	1983	KX060176
19	Human	L	China	2017	MT160087
20	Swine	SER	Germany	1998	JQ743328
21	Swine	KNU-11	South Korea	2011	KC852177
22	Swine	JX/2015/1221/PIV5	China	2015	MT890700
23	Swine	IND/MZ/3013789	India	2015	MW273368
24	Swine	IND/MZ/3013814	India	2015	MW273369
25	Swine	SH/2015/122/PIV5	China	2015	MT890698
26	Swine	HLJ2015/DP1-1/PIV5	China	2015	MT890696
27	Swine	HLJ2015/DP2-1/PIV5	China	2015	MT890697
28	Swine	HuB/YC/2015/PIV5	China	2015	MT890699
29	Swine	SH/2015/1202	China	2015	MK028670
30	Swine	T263	South Korea	2016	MK423233
31	Swine	T361	South Korea	2016	MK423234
32	Swine	T361	South Korea	2016	MK423235
33	Swine	T399	South Korea	2016	MK423236
34	Swine	T434	South Korea	2016	MK423237
35	Swine	Carina(PF-2)	South Korea	2017	MF170888
36	Swine	M32	South Korea	2017	MK423238
37	Swine	M129	South Korea	2017	MK423239
38	Swine	M197	South Korea	2017	MK423240
39	Swine	M293	South Korea	2017	MK423241
40	Swine	Rigel(PF-1)	South Korea	2017	MF170889
41	Swine	N99	South Korea	2018	MK423242
42	Swine	N163	South Korea	2018	MK423243
43	Swine	HLJ	China	2020	OK505006
44	Simian (Cell Culture)	W3A	United States	1964	JQ743318
45	Bovine	CH19-MMH	Switzerland	1998	MN735204
46	Bovine	PV5-BC14	China	2014	KM067467
47	Panthera tigris altaica	PIV5-SR	China	2015	KY685075
48	Panthera tigris tigris	HMZ	China	2018	MH370862
49	Pangolin	PIV5-GD18	China	2017	MG921602
50	Pangolin	CAN	China	2018	MH362816
51	Lesser panda	ZJQ-221	China	2015	KX100034
52	Equine	XJ033	China	2018	MN604146
53	Serpentine	China/HB01/2019	China	2019	MT124463
54	Tick	PIV5/Tick/Heilongjiang/2019	China	2019	MW051776
