# Supplementary material for: An Improved Duplex Real-Time Quantitative RT-PCR Assay with a Canine Endogenous Internal Positive Control for More Sensitive and Reliable Detection of Canine Parainfluenza Virus 5
Source: Vet Sci. 2023 Feb 10;10(2):142. doi: 10.3390/vetsci10020142 (PMC9965950; doi:10.3390/vetsci10020142)
Supplement: Supplementary file 1 [file vetsci-10-00142-s001.zip › Supplementary Table S2.pdf]

Table S2. Comparison of diagnostic results for 16 canine parainfluenza virus 5-positive clinical samples by the newly developed L gene-specific duplex quantitative reverse transcription real-time PCR (dqRT-PCR) assay and previously reported HN gene-specific and N gene-specific qRT-PCR assays.

NO	Sample code	Sample type[a]	Results of different assays (Ct value)			
			dqRT-PCR		qRT-PCR	
			L gene	16s rRNA gene	*HN* gene	*N* gene
1	KNU_C_037	Lung	16.19	17.88	22.17	18.89
2	KNU_C_038	Lung	30.22	16.46	36.19	32.43
3	KNU_C_095	NPS	22.06	15.94	27.58	24.82
4	KNU_C_096	NPS	32.49	19.47	36.64	36.10
5	KNU_C_107	NPS	16.59	13.29	21.50	21.30
6	KNU_C_108	NPS	32.94	17.30	37.27	35.77
7	KNU_C_109	NPS	33.63	19.08	39.10	38.39
8	KNU_C_146	NPS	27.27	16.82	32.68	30.16
9	KNU_C_213	NPS	23.99	18.60	27.69	27.50
10	KNU_C_225	NPS	27.78	20.19	31.00	31.06
11	KNU_C_226	NPS	30.42	24.37	34.65	30.92
12	KNU_C_230	NPS	24.51	20.65	No ct value[b]	28.20
13	KNU_C_261	NPS	32.08	20.78	36.11	35.16
14	KNU_C_271	NPS	22.97	16.01	27.98	24.97
15	KNU_C_352	NPS	32.46	18.38	38.20	34.52
16	KNU_C_359	NPS	32.02	21.57	36.18	34.90
The Ct value range of positive samples			16.19-33.63	13.29-24.37	21.5-39.1	18.89-38.39

[a] NPS, nasopharyngeal swab.

[b] The sample was retested by SYBR Green-based qRT-PCR under the same reaction conditions as previously reported CPIV5 *HN* gene-specific qRT-PCR (Windsor et al., 2006), except that the mismatched probe was excluded, and the result showed that CPIV5 RNA was successfully amplified from the sample by the SYBR Green-based qRT-PCR with a Ct value of 25.81.
